# Evaluation of Response to Therapy in a Patient with Lung Cancer: Correlation of Sclerotic Bone Lesions with F 18 FDG PET/CT and Bone Scintigraphy

**DOI:** 10.4274/MIRT.20.06

**Published:** 2011-04-01

**Authors:** Tamer Özülker, Filiz Özülker, Aysun Küçüköz Uzun, Tarık Tatoğlu, Tevfik Özpaçacı

**Affiliations:** 1 Okmeydani Training and Research Hospital, Department of Nuclear Medicine, Istanbul, Turkey

**Keywords:** Positron emission tomography; bone scintigraphy; bone metastases; medical oncology

## Abstract

A 64-year-old male patient with small cell lung cancer underwent Fluorine-18 fluorodeoxyglucose (F 18 FDG) positron emission tomography (PET)/CT scan which revealed multiple F 18 FDG uptake in the spine, both humeri, ribs, pelvis and proximal long bones. There was no obvious lytic or sclerotic bone destruction accompanying these lesions on CT component of the study. After the patient received six courses of chemotherapy a repeat F 18 FDG-PET/CT was performed for evaluation of therapy response. The PET/CT showed the presence of multiple sclerotic lesions on CT without FDG uptake, corresponding to the bone lesions on the previous PET/CT scan. A concomitant Tc 99m Methylene diphosphonate (Tc 99m MDP) bone scintigraphy (BS) revealed no pathologically increased Tc 99m MDP uptake in the skeletal system. The FDG avid lesions in the skeletal system, which were not sclerotic initially, were transformed into FDG non-avid sclerotic lesions after chemotherapy. This was attributed to the direct effect of previous successful therapy for bone metastases, leading to the transformation of metabolically active disease, into blastic metabolically inactive metastases. In conclusion, a F 18 FDG negative bone lesion, which is sclerotic on CT, may represent post-treatment osteoblastic change rather than active tumor and BS might play a role in the discrimination of these two situations.

**Conflict of interest:**None declared.

## INTRODUCTION

In patients with malignancies that potentially metastasize to bone, the early diagnosis of bone metastasis is crucial to determine the prognosis and to plan the therapy (1,2). The purpose of imaging is to identify early bone metastasis, to determine the full extent of the skeletal involvement, to assess the presence of accompanying complications, like fractures and cord compression, to monitor response to therapy and to guide biopsy in case histological confirmation is indicated (2,3,4). Early detection of metastatic disease may prevent these complications and make it easier to control the disease process. Identification of the tumor mass at an early stage will increase the survey and offer a better quality of life. Tc 99m MDP based skeletal scintigraphy has been the standard method for the initial staging of bone tumors. However, it detects bone metastases at a relatively advanced stage of tumor infiltration, only after osteoblastic host reaction to tumor deposits has begun. In addition, anatomical detail, sensitivity, and specificity are limited (2,3,4,5). BS is not found suitable for evaluating the therapy response because of these factors and the confounding effect of flare phenomenon after therapy (6). Bone metastasis is present in 20-30% of patients at the initial diagnosis of lung cancer (7,8,9) and BS has been used to diagnose bone metastases in lung cancer. More recently, F 18 FDG-PET/CT imaging, which has various oncological applications, has been recommended as an important complementary tool to BS in the detection of bone metastases and evaluation of therapy response. 

Here we present a case showing the importance of the use of F 18 FDG-PET/CT scan and BS together in the evaluation of treatment response and detection of bone metastases. 

## CASE

**Figure 1:**
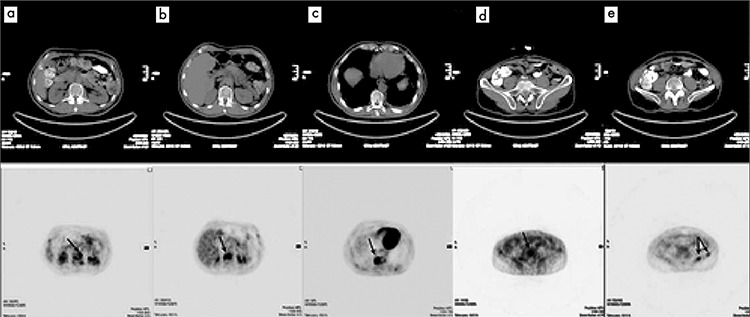
Selected axial slices of PET and CT images from F 18 FDG PET/CT scan showing pathologically increased FDG uptake at dorsal vertebrae (a, b, c), fifth lumber vertebra (d) and left iliac wing (e) without any corresponding lytic or sclerotic lesion on CT images

**Figure 2:**
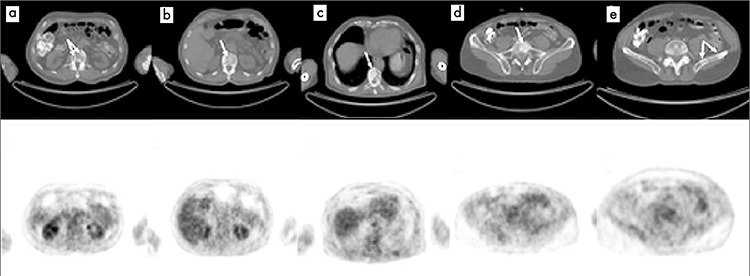
Selected axial slices of PET and CT images from F 18 FDG PET/CT scan obtained after the patient received chemotherapy showing osteblastic lesions at dorsal vertebrae (a, b, c), fifth lumber vertebra (d) and left iliac wing (e) on CT images without FDG avidity

**Figure 3:**
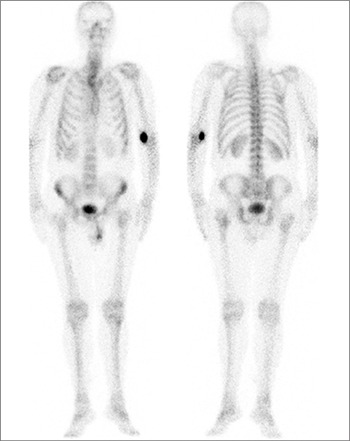
Tc 99m MDP bone scintigraphy, performed after the patient had undergone chemotherapy, showing no pathologically increased Tc 99m MDP accumulation

A 64-year-old male patient consulted with the complaint of chronic cough, unresponsive to medication and lasting for more than four weeks, in July 2009. He had a history of smoking with one package a day for 30 years. In chest X-ray a large soft tissue mass centrally located in left lung was detected and thoracic computerized tomography (CT) revealed a left hilar mass in soft tissue density, surrounding left main bronchus and descending aorta. The mass was invading mediastinum with extension to subcarinal region. There were also multiple parenchymal nodules in both lungs and enlarged N2, N3 lymph nodes consistent with metastatic involvement. Histopathological findings after transbronchial biopsy revealed small cell lung cancer. Synchronously F 18 FDG PET/CT scan was done in order to identify any possible distant metastasis and complete the staging procedure. PET/CT study was performed using an integrated PET/CT scanner which consisted of a full-ring HI-REZ LSO PET and a 6-slice CT (Siemens Biograph 6, Chicago, USA). Patient was instructed to fast for at least 6 h before F 18 FDG injection. The patient was injected 555 MBq F 18 FDG. After 50 minutes of waiting in a semireclined relaxed chair, the patient was imaged using an integrated PET/CT scanner. The CT portion of the study was done without an iv contrast medium, just for defining anatomical landmarks and making attenuation correction on PET images. CT was acquired first with the following parameters: 50 mAs, 140 kV and 5-mm section thickness. Whole-body CT was performed in a craniocaudal direction. PET images were acquired on a 3D mode, from the base of the skull to the mid-thigh, with five to seven bed positions of 3 min each and PET data were collected in a caudocranial direction. The CT data were matched and fused with the PET data. Multiple metastatic lesions showing moderate to high F 18 FDG uptake in the spine, both humeri, ribs, pelvis and proximal long bones were detected on PET/CT, in addition to the high FDG uptake in the left lung mass, parenchymal nodules and mediastinal lymph nodes. There was no obvious lytic or sclerotic bone destruction accompanying these lesions on CT component of the study (Figure 1). After the patient received six courses of chemotherapy, a repeat PET/CT study was performed for evaluation of therapy response in December 2009. PET/CT showed the presence of multiple sclerotic lesions on CT without FDG uptake, corresponding to the bone lesions on the previous PET/CT scan. Relatively decreased FDG uptake, compared to the first study, was detected in the mass lesion in the left lung, parenchymal nodules and mediastinal lymph nodes reflecting a partial remission after therapy (Figure 2). A concomitant (BS) was performed using a double-head gamma camera (ECam, Siemens Medical Solutions) equipped with low- energy, high-resolution collimators. Whole-body image was obtained 3 to 4 hours after the intravenous injection of 740 MBq (20 mCi) of- Tc 99m MDP at a scan speed of 15 cm/min in the anterior and posterior projection. BS revealed no pathologically increased Tc 99m MDP uptake in the skeletal system (Figure 3). Informed consent was obtained from the patient.

## DISCUSSION

It is a well known fact that F 18 FDG PET is more sensitive in detecting lytic metastases than sclerotic metastases. Cook et al (10) demonstrated in their study with 23 breast cancer patients who had progressive bone metastases, that in the subgroup of patients with blastic bone metastases, F 18 FDG PET detected fewer bone metastases than BS. Similarly, Metser et al. (11) reported an increased F 18 FDG uptake in 100% of metastases presenting as lytic lesions on the CT part of the PET/CT study and in 88% of the metastases presenting as sclerotic lesions. Uematsu et al (12), in a study of 15 patients with breast cancer, showed that F 18 FDG PET was less sensitive than bone scanning in detecting osteoblastic metastases. Nakai et al (13) in a study of 55 breast cancer patients showed that FDG PET was very sensitive (100%) in the detection of lytic lesions, but significantly less sensitive (56%) in the detection of blastic lesions compared with bone scintigraphy (100%). The mechanisms of the higher sensitivity of F 18 FDG PET/CT in detecting lytic metastases are not very well established yet, but it might be because of higher glycolytic rate in this type of metastasis. Sclerotic metastases are relatively hypocellular and consequently lower volumes of viable tumor tissue within individual lesions may have an impact on the degree of uptake of F 18 FDG. In addition, aggressive nature and rapid growth of lytic lesions may outstrip their blood supply, rendering the tumor relatively hypoxic which is known to increase the FDG uptake (15). FDG non-avid osteoblastic lesions can be detected as sclerotic lesions on CT part of the PET/CT studies. Israel et al (15) reported that the discrepancy in anatomical and functional imaging of sclerotic bone metastases could be attributed to previous treatment, which might transform previously lytic skeletal lesions to FDG non-avid sclerotic lesions. They found that PET and CT have a similar detectability rate for bone metastases in untreated patients of 91% and 94% respectively, but in patients who had previously received therapy, however, the detection rate of bone metastases on CT was 96% as compared to 47% on FDG PET. They postulated in their study that FDG PET may become the tool to differentiate between clinically significant and “burnt-out” bone metastases detected on CT component of PET/CT imaging. Similarly, Due et al (16) investigated the clinical relevance of F 18 FDG uptake features of bone metastases with various radiographic appearances by monitoring bone metastases with sequential F 18 FDG-PET/CT imaging in breast cancer patients. They included 25 patients with 146 bone metastases in their study and the majority of the osteolytic (93.5%) and mixed-pattern lesions (81.8%), but fewer of the osteoblastic lesions (61%), showed increased F 18 FDG uptake. After treatment, 80.5% of osteolytic lesions became FDG negative and osteoblastic on CT and 52% of the FDG avid osteoblastic lesions became FDG non-avid. Based on these results, the authors suggested that FDG-negative lesions, which are for the greater part osteoblastic, are more likely to represent post-treatment osteoblastic change rather than active tumor. In our case also, the FDG avid lesions in the skeletal system, which were not sclerotic initially, were transformed into FDG non-avid sclerotic lesions after chemotherapy, suggesting a direct effect of previous successful therapy for bone metastases, leading to the transformation of metabolically active disease, into blastic metabolically inactive metastases. Huyge et al (17) reported a patient with breast cancer in whom serial PET/CT images showed progressive blastic bone metastases on the CT without FDG uptake. These lesions were confirmed as metastases by Tc 99m MDP bone single photon emission computed tomography and steadily rising tumor marker levels. In the present case BS of the patient revealed no metastatic lesions and when its high sensitivity in detecting sclerotic type bone metastases is considered, efficacy of the BS in making discrimination between metastatic and metabolically inactive lesions should be emphasized. A negative BS, makes it possible to rule out metastasis in a sclerotic FDG non-avid lesion, but we must be cautious when making a diagnosis of metastasis depending on a positive bone scan, since the osteoblastic response identified on bone scan persists for some considerable time and the scan therefore remains positive even if there is good response to treatment. The flare response, typically observed in the first few months after successful treatment also makes BS alone an unsuitable way to evaluate treatment response (6). 

In lung cancer, lytic bone metastases are the most common type of metastases. If a skeletal metastasis is confined to bone marrow, F 18 FDG can detect the metastasis at an early stage before osteoblastic response in surrounding bone can be detected on BS. Integrated F 18 FDG-PET/CT was found to be superior to BS in the detection of osteolytic bone metastasis in lung cancer (18-19,22). There has been several other studies comparing the efficacy of conventional BS and FDG PET scans in the detection of bone metastases of various cancers and although the diagnostic results of FDG PET and BS would be different according to the type of primary cancers in detection of bone metastases, FDG PET has been recommended as a substitute for BS in the evaluation of bone metastasis, except for high risk cases with breast and prostate cancers or diabetic patients (20,21,22,23,24,25,26,27,28,29).

Addition of CT with integrated PET/CT systems not only facilitated the anatomical localization of the lesions, but also increased the specificity and sensitivity of the PET study in evaluating the bone lesions as well (16,30). But as we see in our case, BS, despite the abovementioned drawbacks, still plays an important role in the differentiation between metastatic and metabolically inactive bone lesions. 

In this case, we have demonstrated that an F 18 FDG negative bone lesion, which is sclerotic on CT, may represent post-treatment osteoblastic change rather than active tumor and BS has a role in the discrimination of these two situations.
